# Supplementary material for: Crossbred Sows Fed a Western Diet during Pre-Gestation, Gestation, Lactation, and Post-Lactation Periods Develop Signs of Lean Metabolic Syndrome That Are Partially Attenuated by Spirulina Supplementation
Source: Nutrients. 2022 Aug 30;14(17):3574. doi: 10.3390/nu14173574 (PMC9460909; doi:10.3390/nu14173574)
Supplement: Supplementary file 1 [file nutrients-14-03574-s001.zip › nutrients-1854203-supplementary.pdf]

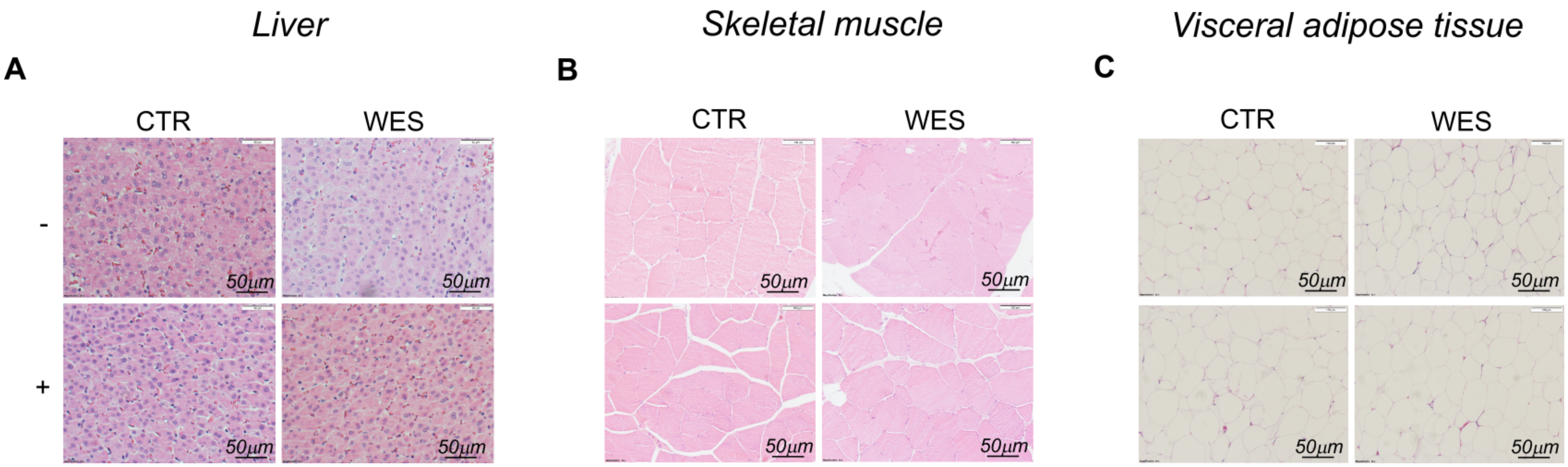

SUPPLEMENTARY FIGURE S1. Morphology of liver, skeletal muscle and visceral adipose tissue (VAT) collected at slaughter. Representative images of A) liver, B) skeletal muscle and C) VAT histology.

CTR: Control diet; WES: Western diet; –: non-supplemented; +: spirulina-supplemented.

SUPPLEMENTARY TABLE S1.

Effects of Western diet and spirulina supplementation on the FA composition of liver and skeletal muscle of sows (g/100g total FA)[§].

Diet (D)		CTR		WES			*Significance*		
Spirulina (Sp)		–	+	–	+	SEM	D	Sp	D×Sp
	Tissue								
C14:0	Liver	0.41	0.27	0.29	0.44	0.138	*n.s.*	*n.s.*	#
	Muscle	1.26	1.26	1.30	1.24	0.050	*n.s.*	*n.s.*	*n.s.*
C16:0	Liver	14.8	14.9	14.4	15.2	0.95	*n.s.*	*n.s.*	*n.s.*
	Muscle	24.6	24.0	24.3	24.3	0.59	*n.s.*	*n.s.*	*n.s.*
iso C16:0	Liver	0.39	0.29	0.31	0.47	0.114	*n.s.*	*n.s.*	#
	Muscle	0.38	0.37	0.41	0.39	0.022	*n.s.*	*n.s.*	*n.s.*
C16:1 *n*–7	Liver	0.78	0.62	0.79	0.95	0.270	*n.s.*	*n.s.*	*n.s.*
	Muscle	2.67	2.75	2.69	2.83	0.256	*n.s.*	*n.s.*	*n.s.*
C17:0	Liver	0.54	0.45	0.54	0.23	0.096	*n.s.*	*	*n.s.*
	Muscle	0.14^{a}	0.14^{a}	0.17^{b}	0.13^{a}	0.008	*	*	**
C18:0	Liver	25.1	25.1	27.4	26.3	1.61	*n.s.*	*n.s.*	*n.s.*
	Muscle	14.6	13.8	16.1	14.3	0.66	*	**	*n.s.*
C18:1 *cis*–9	Liver	15.4	14.1	15.0	18.1	2.77	*n.s.*	*n.s.*	*n.s.*
	Muscle	39.1	39.4	37.6	40.0	1.84	*n.s.*	*n.s.*	*n.s.*
C18:1 *cis*–11	Liver	n.a	n.a.	n.a.	n.a.	n.a.	*n.a.*	*n.a.*	*n.a.*
	Muscle	3.43	3.72	3.14	3.47	0.203	#	#	*n.s.*
C18:2 *n*–6 (LA)	Liver	15.8	15.2	13.3	12.4	0.86	***	*n.s.*	*n.s.*
	Muscle	9.76	9.84	9.46	7.75	1.620	#	*n.s.*	*n.s.*
C18:3 *n*–6 (GLA)	Liver	0.26	0.34	0.24	0.36	0.059	*n.s.*	#	*n.s.*
	Muscle	0.04	0.04	0.05	0.05	0.008	*n.s.*	*n.s.*	*n.s.*
C18:3 *n*–3 (ALA)	Liver	0.16	0.14	0.16	0.21	0.099	*n.s.*	*n.s.*	*n.s.*
	Muscle	0.21	0.26	0.28	0.23	0.008	*n.s.*	*n.s.*	*
C20:0	Liver	0.18	0.17	0.12	0.10	0.068	*	*n.s.*	*n.s.*
	Muscle	0.39	0.32	0.30	0.30	0.082	#	#	*n.s.*
C20:1 *n*–9	Liver	0.32	0.33	0.26	0.25	0.023	***	*n.s.*	*n.s.*
	Muscle	0.83	0.84	0.71	0.75	0.067	**	*n.s.*	*n.s.*
C20:2 *n*–6	Liver	0.45	0.45	0.24	0.20	0.047	***	*n.s.*	*n.s.*
	Muscle	0.36	0.36	0.23	0.22	0.028	***	*n.s.*	*n.s.*
C20:3 *n*–6 (DGLA)	Liver	0.55	0.51	0.71	0.53	0.106	*	*	*n.s.*
	Muscle	0.15	0.17	0.18	0.15	0.038	*n.s*	*n.s.*	*n.s.*
C20:4 *n*–6	Liver	17.5	19.2	18.3	17.5	1.67	*n.s.*	*n.s.*	*n.s.*
	Muscle	1.27	1.47	2.23	1.60	0.681	*n.s.*	*n.s.*	*n.s.*
C20:5 *n*–3 (EPA)	Liver	0.26	0.22	0.51	0.43	0.048	***	*n.s.*	*n.s.*
	Muscle	0.11	0.11	0.16	0.12	0.098	*n.s.*	*n.s.*	*n.s.*
C22:4 *n*–6	Liver	1.27	1.43	0.97	0.83	0.115	***	*n.s.*	*n.s.*
	Muscle	0.08	0.12	0.10	0.08	0.068	*n.s.*	*n.s.*	*n.s.*
C22:5 *n*–3	Liver	1.59	1.82	1.70	1.38	0.223	*n.s.*	*n.s.*	*n.s.*
	Muscle	0.14	0.15	0.21	0.16	0.055	*n.s.*	*n.s.*	*n.s.*
C22:6 *n*–3	Liver	1.47	1.36	1.45	1.45	0.394	*n.s.*	*n.s.*	*n.s.*
	Muscle	0.04	0.04	0.07	0.06	0.021	#	*n.s.*	*n.s.*
C22:5 *n*–6	Liver	0.38	0.28	0.32	0.21	0.130	*n.s.*	#	*n.s.*
	Muscle	n.a.	n.a.	n.a.	n.a.	n.a.	*n.a.*	*n.a.*	*n.a.*

CTR: Control diet; WES: Western diet; n.a.: not analysed.

Data are presented as least square mean ± standard error of the mean (SEM). Within a row, least squares means without a common superscript differ (*p* <.05). Statistical significances were set at **p* <.05, ** *p*<.01, ****p* <.001. Trends were defined as #.05 < *p* <.10. n.s.: not significant.

[§]Only fatty acids (FA) with a proportion > 0.2 g/100 g total FA in at least one of the groups or tissues are displayed.
